# Four Birds with One Stone: Enhancing Integrated Current Density beyond 25.5 mA cm^–2^ in Perovskite Solar Cells through Advanced Fabrication Strategies

**DOI:** 10.1021/acsaem.5c02387

**Published:** 2025-10-16

**Authors:** Luigi Angelo Castriotta, Erica Magliano, Maurizio Stefanelli, Sathy Harshavardhan Reddy, Daimiota Takhellambam, Marco Luce, David Becerril Rodriguez, Antonio Cricenti, Francesco Di Giacomo, Matteo Cirillo, Luigi Vesce, Aldo Di Carlo

**Affiliations:** † CHOSE − Centre for Hybrid and Organic Solar Energy, Department of Electronic Engineering, 9318 University of Rome ‘‘Tor Vergata’’, via del Politecnico 1, 00133, Rome, Italy; ‡ Rayleigh Solar Tech, 1 Research Dr, Dartmouth, NS B2Y 4M9, Canada; § 204549 Istituto di Struttura della Materia- Consiglio Nazionale delle Ricerche Roma (ISM-CNR), via del Fosso del Cavaliere 100, 00133, Rome, Italy; ∥ Solertix, via Eusebio Chini 15, 00147, Rome, Italy; ⊥ Department of Physics, University of Rome “Tor Vergata”, Via della Ricerca Scientifica 1, 00133 Rome, Italy; # Department of Biomedicine and Prevention, University of Rome “Tor Vergata”, Via Montpellier 1, 00133 Rome, Italy

**Keywords:** Perovskite Solar Cells, FAPbI_3_, Integrated Current Density, Additive Engineering, Passivation, Annealing, Substrate Optimization

## Abstract

Perovskite solar technology stands at a pivotal juncture, marked by remarkable efficiencies reaching up to 27.3%. However, these achievements remain confined to a handful of research institutions globally, primarily attributed to the inherent challenge of stabilizing the narrow bandgap structure of FAPbI_3_-based perovskite. This study delves into the exploration of various strategies to enhance the performance of FAPbI_3_-based perovskite. Specifically, it scrutinizes the impact of substrate selection, annealing processes, additive incorporation, and passivation strategies. Through systematic investigation and meticulous refinement of these key parameters, we were able to obtain optimized device performance with an integrated current density of 25.59 mA cm^–2^ and a power conversion efficiency up to 21.86%, starting from a reference device of 23.11 mA cm^–2^ and 18.23% values for integrated current density and device efficiency, respectively. This work endeavors to propel the broader adoption and practical realization of high-performance perovskite solar technologies, thereby contributing to the sustainable advancement of renewable energy solutions.

## Graphical abstract

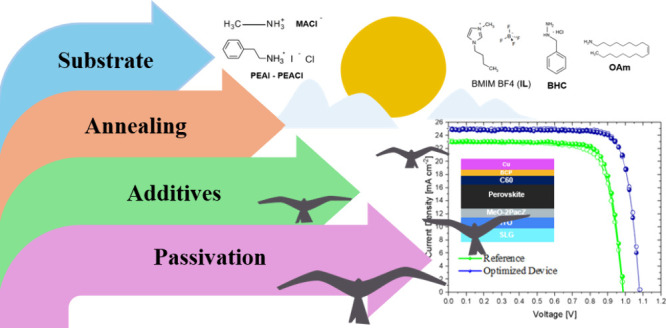

## Introduction

Perovskite solar technology has emerged as a promising force in the renewable energy sector and is lauded for its potential to reshape the solar photovoltaic landscape. Achieving efficiencies of up to 27.3%, perovskite solar cells have demonstrated a capacity to rival and even surpass traditional silicon-based counterparts, marking a significant milestone in the advancement of renewable energy technologies.^1,2^ However, these remarkable achievements remain largely concentrated within a select few research institutions globally, highlighting the enduring challenges in fully harnessing the transformative capabilities of perovskite solar technology. Central to these challenges is the complex nature of FAPbI_3_-based perovskite, characterized by its narrow bandgap structure, which possesses substantial obstacles in terms of stability and reliability. Indeed under ambient conditions, the photoactive black α phase easily converts to the yellow δ phase, leading to poor crystallinity in the FAPbI_3_ film even after high-temperature annealing.^3^ Although several research strategies have been reported to stabilize α-FAPbI3 perovskite,^4^ often structural variation or influence on the intrinsic properties is induced,^5^ broadening the bandgap and unstable crystalline structure, which degrade the device performance.^6^

Despite the promising prospects offered by perovskite solar cells, their journey toward widespread adoption and commercialization is hindered by a variety of complexities, necessitating a concerted effort to overcome key barriers and unlock their full potential. In response to these challenges, our study embarks on an exhaustive exploration of advanced fabrication strategies aimed at enhancing the performance of FAPbI_3_-based perovskite solar cells. At the heart of our investigation lie four crucial parameters: substrate selection, annealing processes, additive incorporation, and passivation strategies. Through a methodical and rigorous approach, we seek to unravel the intricate interplay between these factors and their collective impact on device efficiency, with the overarching objective of surpassing the threshold of 25.5 mA cm ^–2^ in integrated current density. The significance of substrate choice cannot be overstated in optimizing the performance of perovskite solar cells, particularly in terms of minimizing uncovered light absorption and maximizing photon capture. The selection of appropriate substrate material plays an essential role in facilitating efficient charge transport and minimizing losses, demonstrating a profound influence on overall device performance. Equally critical is the role of annealing processes in shaping the structural and morphological characteristics of perovskite films. In addition to solution-processed approaches, other classes of photovoltaic materials prepared by high-temperature solid-state reactions have been extensively studied, including oxide, chalcogenide, and nitride systems. These works (e.g., refs 7–13 ) demonstrate robust phase stability and long-term performance but often require high processing temperatures, limiting compatibility with flexible or tandem applications. In contrast, our work emphasizes low-temperature strategies for FAPbI_3_, providing complementary insights for researchers interested in both solid-state and solution-processed systems. Alternative fabrication methods, such as physical vapor deposition, chemical vapor deposition, and blade-coating techniques, have also been reported for perovskites and related semiconductors (refs 14–16 ). Including these approaches in the broader landscape highlights the unique advantages of our solution-processed route. Through controlled annealing at temperatures below 150 °C, we aim to mitigate crack formation and ensure the uniformity and integrity of the perovskite layer. This meticulous approach enhances device reliability and contributes to the longevity and stability of perovskite solar cells, addressing a key bottleneck in their commercialization pathway. Additionally, the integration of additives holds immense promise in augmenting the performance of perovskite solar cells by facilitating perovskite crystallization and optimizing charge extraction mechanisms. By selecting and incorporating suitable additives, we aim to enhance the structural uniformity and electronic properties of perovskite films, thereby improving the device efficiency and stability. In parallel, passivation strategies offer a viable pathway toward enhancing the stability and reliability of perovskite solar cells by mitigating interface recombination and minimizing defect density. Through the implementation of passivation layers and surface treatments, we aim to suppress nonradiative recombination processes and enhance charge carrier lifetimes, thereby improving device performance and stability. By systematically optimizing these key fabrication parameters, our study achieves significant advancements in the performance of FAPbI_3_-based perovskite solar cells, resulting in an integrated current density exceeding 25.5 mA cm^–2^ and a power conversion efficiency of up to 21.86% starting from a reference stack delivering 23.11 mA cm^–2^ and 18.23% on integrated current density and device efficiency, respectively. Despite progress, no prior work has systematically optimized FAPbI_3_ devices across four fabrication parameters simultaneously: (i) substrate selection, (ii) annealing protocol, (iii) additive incorporation, and (iv) surface passivation. This gap motivates our study, whose specific objectives are (1) to compare commonly used FTO with ITO substrates and quantify parasitic absorption losses; (2) to identify an annealing window below 150 °C that preserves both perovskite and SAM (self-assembly monolayers) integrity; (3) to evaluate the synergistic role of multiple additives in defect passivation and α-phase stabilization; and (4) to assess the effect of surface passivation on defect suppression and crystallization quality.

Substrate selection is a fundamental consideration in the fabrication of perovskite solar cells, influencing the device performance and stability. The choice of substrate material impacts light absorption, charge transport, and overall device efficiency. Different substrates offer varying mechanical, optical, and electrical properties, which can significantly affect the morphology and crystallinity of the perovskite layer.

The most used rigid substrates are based on fluorine-doped tin oxide (SnO_2_) (FTO) or indium-doped SnO_2_ (ITO). FTO-based substrates can show improved conductivity, despite usually exhibiting a reduced weighted average absorptance (Awt) and an elevated surface roughness.^17^ Conversely, a smooth surface and an improved absorption are shown in the case of ITO-based substrates, even though they display a higher sheet resistance. Here, we evaluated a glass/FTO substrate TEC 7 from Pilkington (7 Ω sq^–1^) and two types of glass/ITO from Kintec with different sheet resistance (10 Ω sq^–1^, denoted as ITO 1, and 20 Ω sq^–1^, indicated as ITO 2), as well as transmittance, as shown in Figure 1a. Glass-FTO substrates were fabricated as a reference baseline, reflecting their widespread use in perovskite solar cells (PSCs) research. This comparison with ITO substrates highlights the specific optical and morphological advantages of ITO. The ITO substrates exhibit a smooth surface, as demonstrated by the low surface root-mean-square (rms) roughness, Sq, measured with AFM, showing a value of (4.34 ± 0.25) nm (see Figure 1b, c). A significantly higher Sq of (30.1 ± 3.8) nm was observed in the FTO case, where a more irregular surface is evident from the height image in Figure 1d, e and Figures S2 and S3. An increased surface roughness of the substrate can enhance light trapping and reduce reflection, even though the subsequent layer deposition can be challenging. This is particularly true for the p-i-n configuration, where ultrathin HTLs (hole transport layers) are typically employed, i.e. self-assembled monolayers.^18^ Therefore, uncovered areas or disuniformity could easily form, resulting in shunt paths. Additionally, rough or textured surfaces can induce the formation of agglomeration or clusters of material (HTL and/or perovskite), leading also to irregular optical and electrical properties over the film.^19,20^

**Figure 1:**
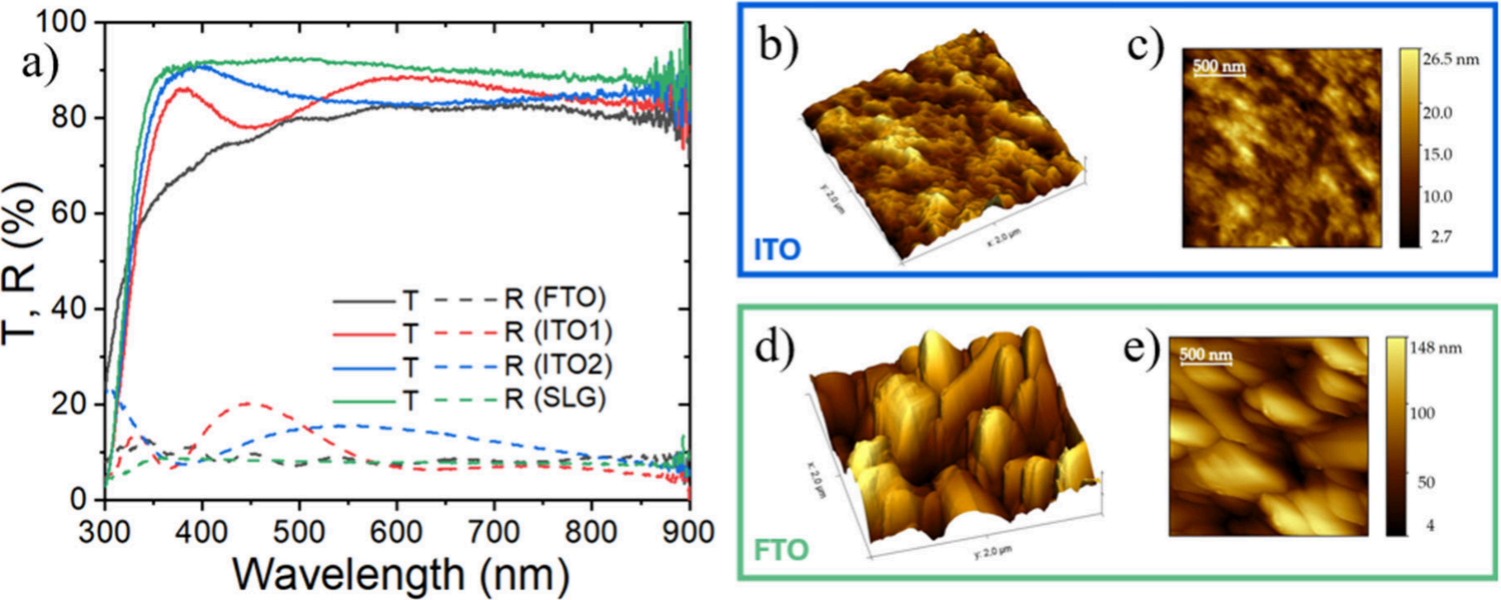
(a) Transmittance spectra of the selected substrate SLG (glass), ITO 1, ITO 2 and FTO. (b, d) 3D AFM height images of ITO and FTO, respectively. (c, e) AFM topography images of ITO and FTO, respectively.

In the superstrate configuration, the spectral region of relevance for optical loss evaluation induced by the substrate is the ultraviolet (UV) range, between 300 and 500 nm. In FTO, a reduction in transmittance is observed in the UV, which could hamper the absorption, as shown in Figure 1a. The transmittance of ITO-2 is the highest in this range, making it a viable solution for mitigating parasitic absorption. On the other hand, ITO-2 exhibited the higher sheet resistance as well, which could increase the series resistance R_S_ of the final device and lower the FF. However, these losses can have a significant impact when converting to large area devices, but they are considered negligible in our investigation on small area. Nonetheless, a possible approach to reduce R_s_ can be followed in the large area case.^21^ Based on these considerations, the optimal trade-off was identified in utilizing ITO-2 substrates. The performance comparison is further supported by J–V with the corresponding parameters (see Figure S1 and Table 1 in the SI), which demonstrate that devices fabricated on ITO-2 exhibit improved short-circuit current density due to reduced parasitic absorption relative to FTO.

**Table 1:** Champion Cell Parameters for the Reference Device and Optimized Device

**Champion Cell**	**Voc [V]**	**Jsc** **[mA cm** ^**–2**^ **]**	**J**_**int**_ **[mA cm** ^**–2**^ **]**	**FF [%]**	**PCE [%]**
Reference	0.98	23.31	23.11	79.81	18.23
Optimized Device	1.06	25.68	25.59	80.32	21.86

The annealing temperature plays an important role in the fabrication process of FAPbI_3_-based perovskite solar cells, giving a significant influence on the structural, morphological, and optoelectronic properties of the perovskite film. FAPbI_3_ perovskite has the inconvenience of phase transition that can occur from the α-phase to the δ-phase spontaneously because it is energetically favored at RT when the α-FAPI is not stabilized or has some phase impurities that drive the phase transition. The delta phase instead, once is formed, can be converted to the α-phase only with temperature > 150 °C.^3^

Many parameters affect or can be affected by the annealing step; the chemistry and composition of the absorbing material can easily change the enthalpy of the crystallization process, affecting the crystal growth and crack formation, which are crucial for achieving high-efficiency solar cells.^22^ Moreover, the annealing temperature directly impacts the degree of ion migration and defect formation within the perovskite film, ultimately affecting charge carrier dynamics and device stability.^23^ Finally, the annealing temperature provides control of phase stability but also can affect the layers underneath the perovskite if they are sensible to high temperatures. In the case of our devices in which SAM are used as ETL (electron transport layer), HTL (hole transport layer) the annealing temperature cannot be used at 150 °C to anneal the absorbing material. To prove this, we tested both NIP and PIN configurations with planar structure (SnO_2_ based) and SAM as ETL and HTL, respectively, with 150 °C annealing temperature for perovskite. From scanning electron microscopy (SEM) investigation, it is clear that reaching 150 °C has a direct impact on the layer’s underneath and on the perovskite morphology consequently. In the case of the NIP structure, perovskite film results compact, with a good crystallinity and no pinholes at the grain boundaries. For the PIN device instead, perovskite shows deep holes alongside the grain boundaries which are supposed to be one of the main reasons for current and voltage leakage on these devices before annealing optimization. All this proves that the morphology and the coverage of the SAM layer after annealing treatment at 150 °C are drastically affected. In the literature, thermogravimetric analysis on MeO-2PACz shows that at 150 °C the material starts to lose weight (less than 5%),^24^ but this can be still impactful on the morphology of the thin film and give us the basement for our speculation on the low crystallinity of the perovskite layer, induced by the SAM degradation.^25^

For these reasons we optimize the annealing temperature under the temperature of 150 °C. Many works in the literature present an efficiency above 21% for devices fabricated at a maximum of 100 °C.^26–28^ Finally we found that at an annealing temperature of 120 °C the perovskite and the SAM film are not affected by any degradation and the efficiency and reproducibility of our devices are improved. SEM images at 150 °C are shown to highlight the degradation of the SAM in PIN structures, whereas at the optimized 120 °C condition, no degradation is observed. The improved crystallinity and absence of pinholes directly correlate with the enhanced efficiency and reproducibility of our devices (see Figure 2).

**Figure 2:**
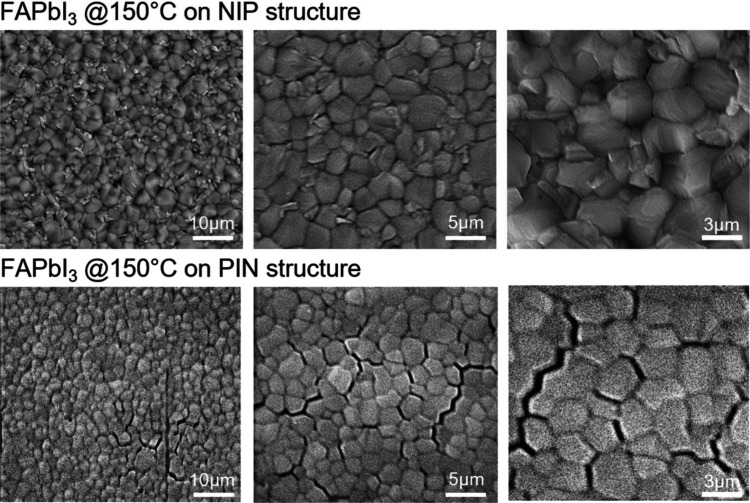
Scanning electron microscopy (SEM) images of FAPbI_3_-based perovskite annealed at 150 °C on the NIP structure (top) and on the PIN structure (bottom). Three different magnifications were taken, with scale bars at 10, 5, and 3 μm on the left, center, and right part of the scheme, respectively.

Additive incorporation and passivation strategies are mainstream aspects in the optimization of perovskite solar cells, with the aim of enhancing device efficiency and stability. Additives are introduced to improve perovskite crystallization and charge transport properties, while passivation strategies focus on reducing defects and enhancing surface properties, to mitigate nonradiative recombination. Through careful selection and integration of additives, as well as implementation of effective passivation techniques, we seek to address key challenges such as grain boundary recombination and interface defects.

Recently, we reported the passivation of defects via introduction of additives like 1-Butyl-3-methylimidazolium tetrafluoroborate (BMIM BF4), Benzyl hydrazine hydrochloride (BHC) and Oleylamine (OAm) in various perovskites to improve the performance and stability.^29^ Similar to the double cation and triple cation perovskites, when these additives are introduced in FAPbI_3_, we saw a similar improvement in the performance. BMIM-BF_4_, BHC, and OAm were chosen because they provide complementary functions in suppressing trap states, modulating surface energy, and passivating defects. Their combination with MACl and PEAI, specifically for stabilizing FAPbI_3_, has not been systematically investigated before this work. Although these additives passivate defects in most perovskites, FAPbI_3_ requires additional additives to stabilize the phase. MACl and PEAI have both shown stabilization of the FAPbI_3_ into the α-phase. The introduction of MACl can help in adjusting the ionic size within the crystal lattice, reducing lattice strain and improving the structural stability of the FAPbI_3_,^30,31^ while the incorporation of large phenylethylammonium cation (PEA+) from the perovskite precursors leads to an interaction with FAPbI_3_ crystals.^32^ This interaction facilitates the formation of the cubic perovskite phase during crystallization. Subsequently, these PEA+ ions functionalize the grain boundaries after the crystallization process is complete. In addition, it is worth noting that the FAPbI_3_ formulations we used contain a 4% excess of the lead iodide in the perovskite inks as it decreases charge trap densities and elongates carrier lifetimes.^31^ All additives (BMIM-BF_4_, BHC, OAm, MACl, and PEAI) were introduced simultaneously into the perovskite precursor solution 30 min before spin-coating, with concentrations specified in the Supporting Information. For the surface passivation, PEACl is a great choice to manage defects at the surface of the perovskite absorber layer.^33^

Figure 3 shows the X-ray diffraction (XRD) patterns of the FAPbI_3_ layers with additives and the passivation layer. The strong peak at 12.8° in the reference XRD graph corresponds to the presence of excess PbI_2_, and a small peak at 11.9° corresponds to the unwanted δ-phase of FAPbI_3_. The XRD patterns for all of the perovskites are not very different. Like our previous work, the addition of additives to FAPbI_3_ has reduced the lead iodide peak intensity, increased the peak intensity of the α-phase present at 14°, and removed the unwanted δ-phase, suggesting that its crystallization is strongly improved. The crystallization improvement (reduced PbI_2_, enhanced α-phase, and suppressed δ-phase) results from the combined addition of BMIM BF_4_, BHC, OAm, MACl, and PEAI, rather than any single additive. In addition, there are no peaks shifting, indicating that the additives passivate mainly the grain boundaries and do not influence the lattice. Similarly, when the PEACl layer was introduced to the reference perovskite, changes similar to those of the sample with additives were observed, which suggests that surface passivation of perovskite also improves the crystallization of the FAPbI_3_. Further, the absence of any peak below 5° confirms that there is no formation of 2D perovskite. Combining both the strategies further reduces the lead iodide peak intensity and forms a higher intensity α-phase peak when compared to all the other samples. The suppression of PbI_2_ and δ-phase peaks (11.9°–12.8°) and enhancement of the α-phase peak (14°) confirm that additives and passivation improve phase purity, consistent with the improved PCE shown in Figure 4. The XRD data clearly reveal the mechanistic role of additives and passivation: (i) suppression of the δ-phase at 11.9°, (ii) reduction of excess PbI_2_ at 12.8°, and (iii) enhancement of the α-phase peak at 14°. These trends directly correlate with the device improvements reported in Figure 4, linking phase purity and crystallization quality to higher current density and PCE. It can be concluded from the data that incorporation of additives and surface passivation strategies can improve the phase of FAPbI_3_, making it purer and with less defects.

**Figure 3:**
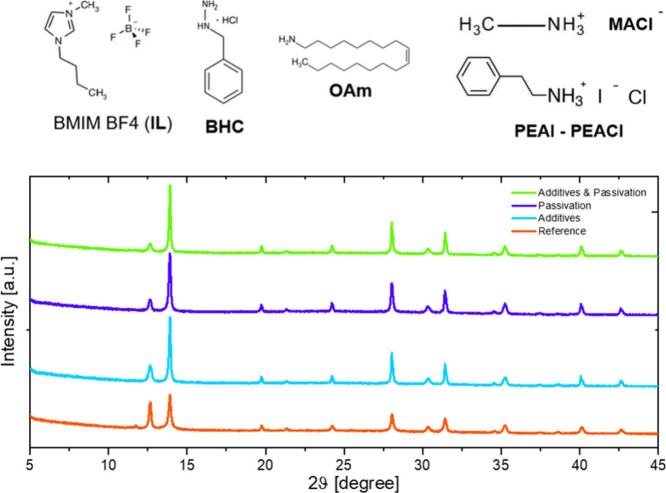
(Top) Additives and passivation molecules used in this work; (Bottom) XRD overview spectra of the perovskite film as it is (reference, red line), with additives only (light blue), with passivation only (violet), and with both additives and passivation (green)

**Figure 4:**
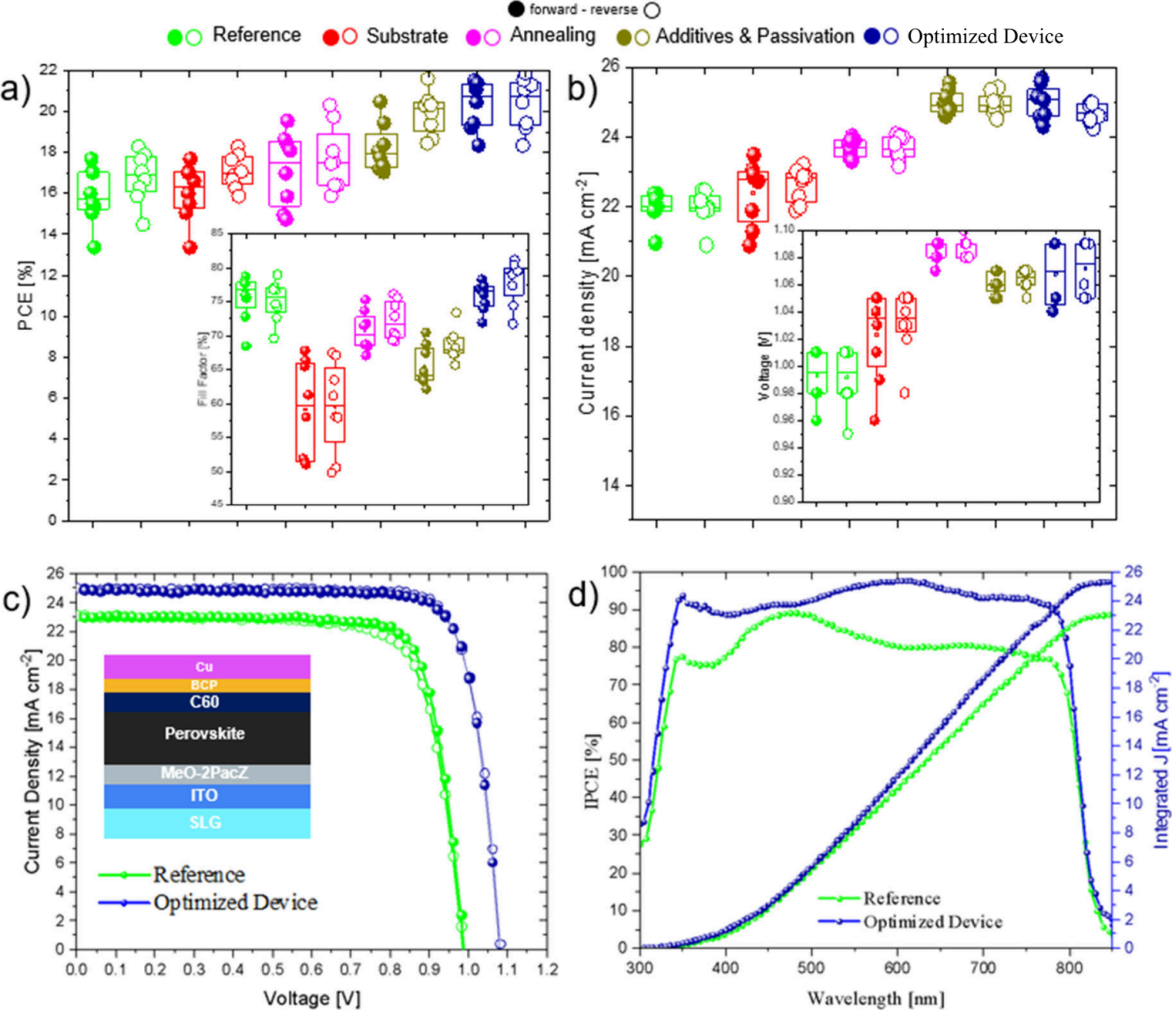
(a and b) Box plot characteristics for the optimized route used in this work, with results showing both forward and reverse scan values; average taken from 8 different devices; (c) J-V curves for reference and optimized device, with the inset of the general device stack used in this work; (d) IPCE characteristics for reference and optimized device, together with the extrapolation of the integrated current density. Blue markers represent devices with 4 birds with 1 stone strategy, showing the cumulative effect of all the strategies.

The combination of those strategies gave us a specific trend in the solar cell device parameters. In particular, Figure 4 shows the main results obtained. Beyond the general defect passivation at grain boundaries, MACl contributes to lattice relaxation by adjusting the ionic size, while PEAI induces surface functionalization that stabilizes the α-phase. This dual mechanism is evidenced in XRD by suppression of the δ-phase and PbI_2_ peaks and enhancement of the α-phase signal at 14°. Such stabilization directly correlates with improved Jsc performance and device reproducibility.

Considering that optimizing a device performance is a multicontrol step contributed by both intrinsic and extrinsic factors, Figure 4 a and b shows the box plots of the various devices optimized through many steps as discussed above. We began with a fair comparison of the substrates making a trade-off between Glass-FTO and Glass-ITO. We can see comparable results between reference devices (Glass-FTO) and Glass-ITO substrate devices. Adding in the annealing factor, which is less than 150 °C, we observe improvement in the device Jsc and Voc, which ultimately leads to higher efficiency. Observing the box plots, the gradual increase of the power conversion efficiency is progressed to higher performance through the addition of additives and the passivation layer. After combining all the four steps of optimization from our gradual study, we finalized our perovskite devices and recorded an increase in the power conversion efficiency from 18.23% (reference device) to 21.86% (optimized device). Figure 4c shows the J-V scan of reference and optimized devices, clearly picturing the increase in overall photovoltaic parameters, which is also shown in Table 1. Our J-V curve is supported by the EQE characteristics in Figure 4d, where we observed an improved performance throughout the entire wavelength range. The extrapolation of the integrated current density also agrees with the values recorded in our J-V curve, which highlights our device performance in boosting the current density from 23.31 mA cm^–2^ (reference device) to 25.68 mA cm^–2^ (optimized devices). As shown in Figure 4a–b, statistical distributions from 8 devices confirm the reproducibility of the optimization strategy, with consistent PCE values around 21–22%. Stability measurements (Figure S4, SI) confirm that optimized devices retain >80% of their initial PCE (T_80_) after 1380 h of continuous illumination at 1 sun equivalent under ambient conditions, underscoring the robustness of our combined strategy.

## Conclusions

In conclusion, this study underscores the critical role of substrate choice in mitigating uncovered light absorption, thereby enhancing the overall device efficiency in perovskite solar cells. Furthermore, our findings emphasize the necessity of meticulous annealing procedures, particularly for PIN structures, where temperatures below 150 °C are optimal to minimize crack formation and preserve device integrity. Additionally, the incorporation of additives and passivation agents has emerged as an indispensable strategy for facilitating perovskite crystallization and optimizing charge extraction mechanisms. Through a systematic approach encompassing these key optimization steps, our research successfully elevates the integrated current density from a reference device of 23.11 mA cm^–2^, up to 25.59 mA cm^–2^ and the PCE from 18.23% to 21.86%, by including those 4 strategies implemented; this represents a substantial advancement in perovskite solar cell performance. While the optimized PCE of 21.86% remains below the world-record values reported for FAPbI_3_ (>25%), the significance of our study lies in demonstrating reproducible and cumulative performance gains through a systematic four-parameter optimization. Although this work focuses on small-area devices, the selected optimization strategies are inherently compatible with scalable fabrication techniques, including blade coating and slot-die deposition. Future work will extend this systematic optimization to larger-area modules. Moving forward, these insights not only contribute to the fundamental understanding of perovskite photovoltaics but also pave the way for the development of more efficient and reliable solar energy technologies, thereby accelerating the transition toward a sustainable energy future.

## Supplementary Material



## Data Availability

The data that support the findings of this study are available on request from the corresponding author. The data is not publicly available due to privacy or ethical restrictions.

## Notes

Additional experimental details, materials and methods, perovskite precursor formulation, device fabrication and characterization procedures, SEM and AFM imaging data, and stability measurements (ISOS-L1) (PDF)
