# Supplementary material for: Four Birds with One Stone: Enhancing Integrated Current Density beyond 25.5 mA cm–2 in Perovskite Solar Cells through Advanced Fabrication Strategies
Source: ACS Appl Energy Mater. 2025 Oct 16;8(20):15043–50. doi: 10.1021/acsaem.5c02387 (PMC12570108; doi:10.1021/acsaem.5c02387)
Supplement: Supplementary file 1 [file ae5c02387_si_001.pdf]

Supporting Information

Four Birds with One Stone: Enhancing Integrated Current Density Beyond 25.5mA cm^{-2} in Perovskite Solar Cells through Advanced Fabrication Strategies

Luigi Angelo Castriotta[1*], Erica Magliano[1], Maurizio Stefanelli[1], Sathy Harshavardhan Reddy[2], Daimiota Takhellambam[1], Marco Luce[3], David Becerril Rodriguez[3], Antonio Cricenti[3], Francesco Di Giacomo[4], Matteo Cirillo[5], Luigi Vesce[1,5,6], Aldo Di Carlo[1,3*]

1. CHOSE – Centre for Hybrid and Organic Solar Energy, Department of Electronic Engineering, University of Rome ''Tor Vergata'', via del Politecnico 1, 00133, Rome, Italy
2. Rayleigh Solar Tech, 1 Research Dr, NS B2Y 4M9, Dartmouth, Canada
3. Istituto di Struttura della Materia- Consiglio Nazionale delle Ricerche Roma (ISM-CNR), via del Fosso del Cavaliere 100, 00133, Rome, Italy
4. Solertix, via Eusebio Chini 15, 00147, Rome, Italy
5. Department of Physics, University of Rome "Tor Vergata", Via della Ricerca Scientifica 1, 00133 Rome, Italy
6. Department of Biomedicine and Prevention, University of Rome "Tor Vergata", Via Montpellier 1, 00133 Rome, Italy

Corresponding author mail address: luigi.angelo.castriotta@uniroma2.it, aldo.dicarlo@cnr.it;

Experimental section

Materials. N,N-dimethylformamide (DMF-anhydrous-Sigma Aldrich), dimethyl sulfoxide (DMSO-anhydrous-Sigma Aldrich), toluene (Sigma Aldrich), chlorobenzene (Sigma Aldrich), C60 (99.50%-Solenne), Bathocuproine (BCP-96%-Sigma Aldrich), Formamidinium iodide (FAI-99.99%-Greatcell solar), Cesium iodide (CsI-99.99%-Sigma Aldrich), Lead Iodide (PbI_2-TCI), were purchased and used without further purification. Additives and passivation agents included 1-butyl-3-methylimidazolium tetrafluoroborate (BMIM-BF_4, Sigma-Aldrich), oleylamine (OAm, Sigma-Aldrich), benzylhydrazine hydrochloride (BHC, Sigma-Aldrich), methylammonium chloride (MACl, Sigma-Aldrich), phenylethylammonium iodide (PEAI, Sigma-Aldrich), and phenylethylammonium chloride (PEACl, Sigma-Aldrich). The self-assembled monolayer (SAM) MeO-2PACz (0.33 mg/mL in ethanol) was used as hole-selective layer. Glass/ITO substrates (10 - 20 Ω sq−1) were purchased from Kintec, Glass/FTO substrates from Pilkington (7 Ω sq-1).

Perovskite solution. $FAPbI_3$-based perovskite precursor solution with a total concentration of 1.45 M was prepared by dissolving 17.92 mg of CsI, 191.64 mg of FAI, and 608.72 mg of PbI_2 in 1 mL of a 4:1 (v/v) DMF:DMSO solvent mixture under stirring. For additive preparation, 50 µL of BMIM-BF_4 was dissolved in 1 mL of DMF, and 50 µL of OAm was separately dissolved in 1 mL of DMF at a concentration of 50 mg/mL. Both solutions were stirred for 30 minutes before use. Thirty minutes prior to deposition, the following were added per 1 mL of perovskite solution: 7% of 0.2 M MACl solution (v/v), 3.45 mg of PEAI, 20 µL of BMIM-BF_4 solution,

10 µL of OAm solution, and 12 µL of BHC solution. The final solution was stirred briefly and filtered using a 0.45 µm PVDF filter before deposition.

Cell fabrication. 2.5 × 2.5 cm^2 glass/ITO or glass/FTO samples were patterned with a UV ps laser (WOP) and diced with a glasscutter (Dyenamo – Stockholm, Sweden). Samples were scrubbed with a water and soap solution (Hellmanex 2% in deionized water) and cleaned with three stages of ultrasonic bath: first in water and soap, then in ultrapure water, and finally in IPA. After drying they were treated for 15 min in a UV/O_3 tool (Novasonic). Samples were immediately transferred into a nitrogen-filled glovebox. 100µl of MeO-2Pacz inks (0.33 mg/ml in ethanol) were first dropped onto the substrate to cover the whole area the solution was spun at 5000 rpm for 20 s and annealed for 10 min at 100 °C. After cooling down, the perovskite ink was spun at 6000 rpm for 35 s, and 150 µL of CB were dropped after 20 s. The film was annealed for 10 min at 100 °C. A 0.45µm PVDF filter was used prior to the deposition. A passivation layer of phenylethylammonium chloride (PEACl, 2 mg/mL in IPA) was spin-coated at 6000 rpm for 25 s and annealed at 100 °C for 5 min. C60, BCP and Cu were deposited by thermal evaporation with a thickness of 28, 7 and 100nm, respectively, using a shadow mask to separate the four cells that are fabricated on each substrate.

Device characterization. Cells were measured with an ABET Sun 2000 class A sun simulator, calibrated with a calibrated Si cell from Rera. The accuracy of the calibration was checked by measuring the internal quantum efficiency of cells (Arkeo system from Cicci Research, Grosseto, Italy), ensuring that the mismatch of the integrated and measured JSC was less than 2%. Data were acquired with a 4-channels source meter unit of Cicci Research with a scan rate of 100 mV s−1 for cells, 300 mV s−1 for modules with three cells.

Imaging characterization. Scanning Electron Microscopy (SEM) experiments were performed by using a TESCAN Analytics VEGA instrument. Atomic Force Microscopy (AFM) measurements were performed with the microscope working in the repulsive regime of contact mode in air at room temperature. Bruker silicon nitride cantilevers MSNL-10 employed Constant force images with a Force of 1 nN have been acquired with a typical scan rate of 2–4 sec/row (400-800 points/row). Data analysis has been performed using the Gwyddion software.

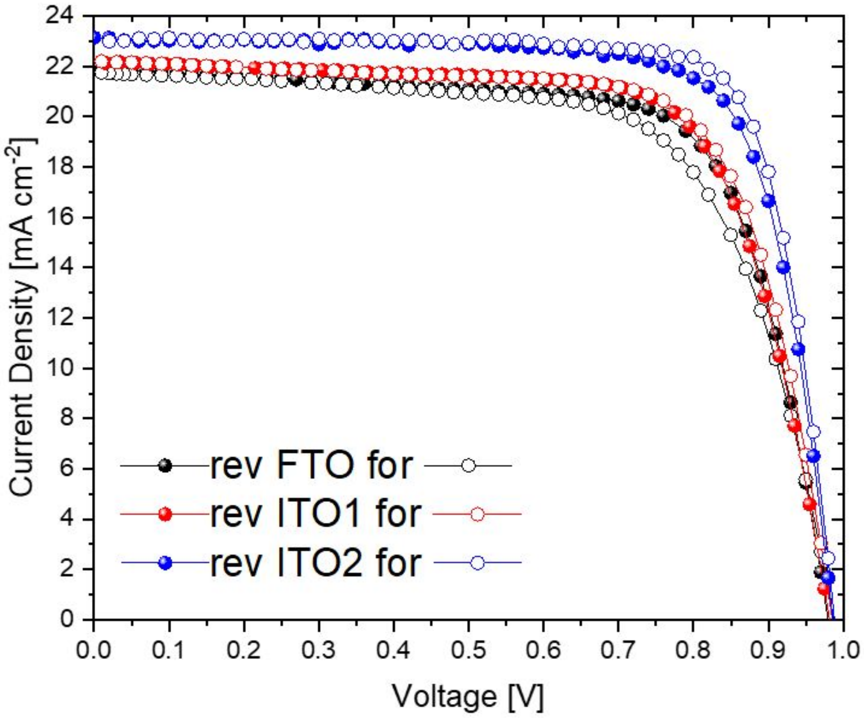

Figure S1. J-V curves for FTO, ITO 10 Ohm sq-1 (ITO1) and ITO 20 Ohm sq-1 (ITO2).

Champion Cell	Voc [V]	Jsc[mA cm^{-2}]	FF [%]	PCE [%]
FTO	0.97	21.85	78.56	16.65
ITO1	0.97	22.29	77.99	16.85
ITO2	0.98	23.31	79.81	18.23

Table 1 Champion cell parameters for FTO, ITO1 and ITO2 based devices.

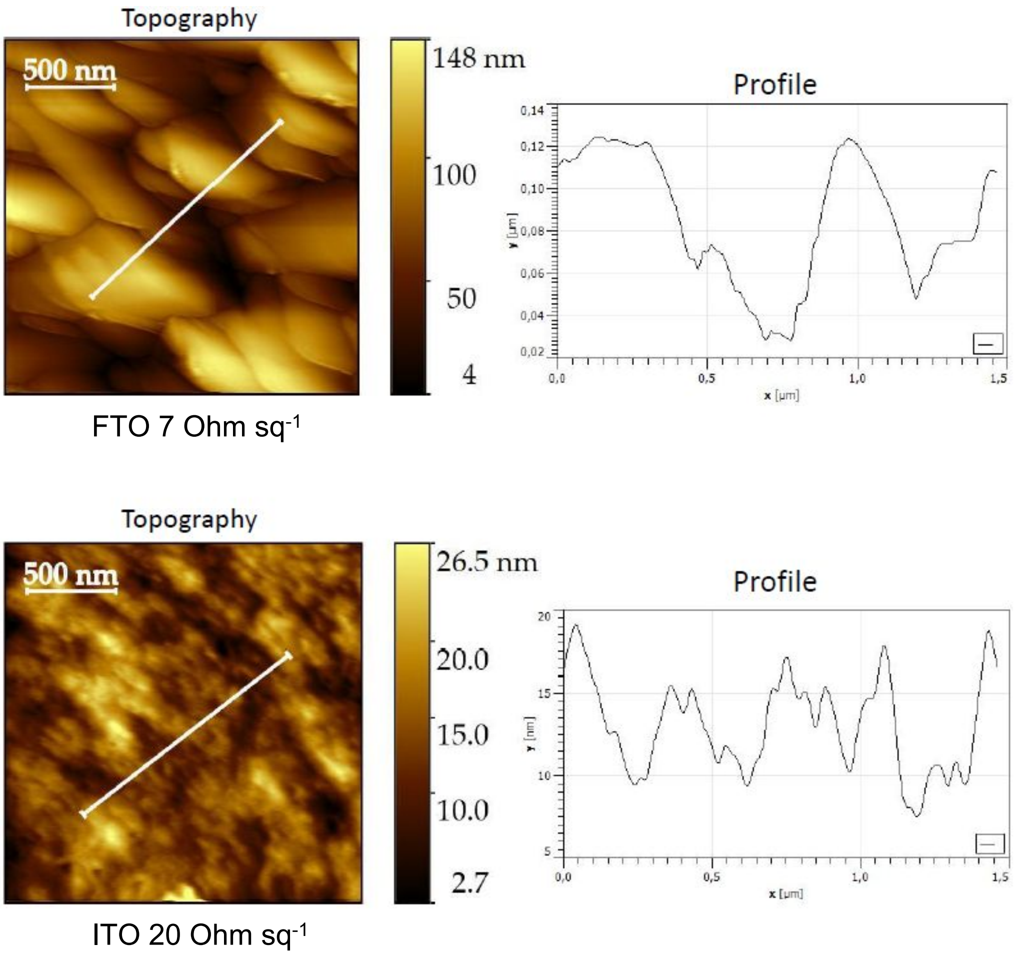

Figure S2. Imaging and profile line roughness analysis of ITO (top) and FTO (bottom) of SLG | TCOs substrates measured by atomic force microscopy (AFM).

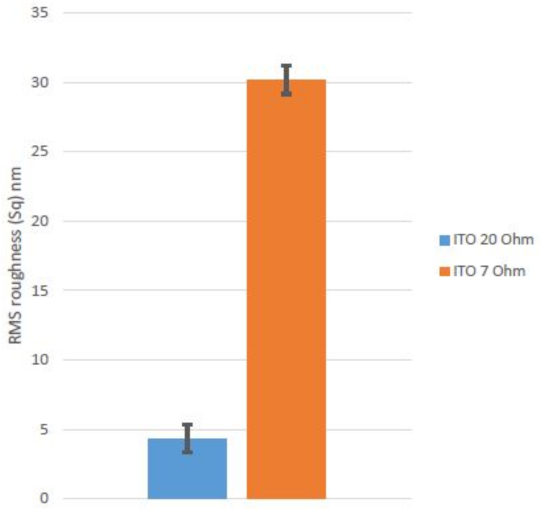

Figure S3. Root mean squared roughness statistics of ITO (blue) and FTO (orange) of SLG | TCOs substrates measured by atomic force microscopy (AFM).

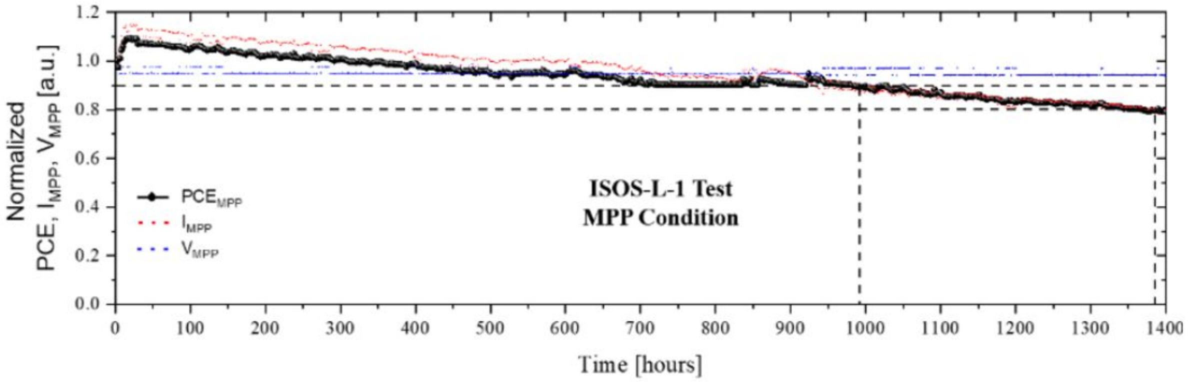

Figure S4. ISOS L1 Test on the best optimized device, with T_{90} and T_{80} reached at 985h and 1380h, respectively.
